# Gene expression profiles in engineered cardiac tissues respond to mechanical loading and inhibition of tyrosine kinases

**DOI:** 10.1002/phy2.78

**Published:** 2013-10-02

**Authors:** Fei Ye, Fangping Yuan, Xiaohong Li, Nigel Cooper, Joseph P Tinney, Bradley B Keller

**Affiliations:** 1 Kosair Charities Pediatric Heart Research Program, Cardiovascular Innovation Institute, University of LouisvilleLouisville, Kentucky; 2 Affiliated Hospital of Guiyang Medical CollegeGuiyang, China; 3 Anatomical Sciences and Neurobiology, University of LouisvilleLouisville, Kentucky

**Keywords:** BIRB796, engineered cardiac tissues, gene expression, mechanical loading, p38MAPK, tyrosine kinases

## Abstract

Engineered cardiac tissues (ECTs) are platforms to investigate cardiomyocyte maturation and functional integration, the feasibility of generating tissues for cardiac repair, and as models for pharmacology and toxicology bioassays. ECTs rapidly mature in vitro to acquire the features of functional cardiac muscle and respond to mechanical load with increased proliferation and maturation. ECTs are now being investigated as platforms for in vitro models for human diseases and for pharmacologic screening for drug toxicities. We tested the hypothesis that global ECT gene expression patterns are complex and sensitive to mechanical loading and tyrosine kinase inhibitors similar to the maturing myocardium. We generated ECTs from day 14.5 rat embryo ventricular cells, as previously published, and then conditioned constructs after 5 days in culture for 48 h with mechanical stretch (5%, 0.5 Hz) and/or the p38 MAPK (p38 mitogen-activated protein kinase) inhibitor BIRB796. RNA was isolated from individual ECTs and assayed using a standard Agilent rat 4 × 44k V3 microarray and Pathway Analysis software for transcript expression fold changes and changes in regulatory molecules and networks. Changes in expression were confirmed by quantitative-polymerase chain reaction (q-PCR) for selected regulatory molecules. At the threshold of a 1.5-fold change in expression, stretch altered 1559 transcripts, versus 1411 for BIRB796, and 1846 for stretch plus BIRB796. As anticipated, top pathways altered in response to these stimuli include cellular development, cellular growth and proliferation; tissue development; cell death, cell signaling, and small molecule biochemistry as well as numerous other pathways. Thus, ECTs display a broad spectrum of altered gene expression in response to mechanical load and/or tyrosine kinase inhibition, reflecting a complex regulation of proliferation, differentiation, and architectural alignment of cardiomyocytes and noncardiomyocytes within ECT.

## Introduction

Following the initial descriptions of generating functional in vitro engineered cardiac tissues (ECTs) composed of immature cardiac cells (Zimmermann et al. 2000; Tobita et al. 2006) there has been a rapid expansion of this robust tissue engineering approach using cells types from a broad range of species including chick, mouse, rat, and human (Zimmermann et al. 2000; Tulloch et al. 2001; Tobita et al. 2006; Lange et al. 2011) and various 3D formulations from microtissues to cell sheets (Nadruz et al. 2005; Zimmermann et al. 2006; Ott et al. 2008; Stevens et al. 2009; Fujimoto et al. 2011; Iyer et al. 2011; Boudou et al. 2012; Masumoto et al. 2012). These ECTs are under active investigation as platforms to optimize in vitro stem cell–derived cardiac tissue maturation toward the goal of cardiac repair and regeneration (Kreutziger and Murry 2011; Zimmermann 2011; Karikkineth and Zimmermann 2013). Although in vitro culture systems cannot fully recapitulate the in vivo maturational environment, ECTs display remarkable cellular remodeling and maturation and can provide insights into the functional and molecular adaptation of the developing and neonatal myocardium (Clause et al. 2009; Lesman et al. 2010; Tiburcy et al. 2011; Zimmermann 2011). ECTs are also now being used as platforms for pharmacologic testing using normal maturing cells and/or reprogrammed stem cells derived from patients with defined cardiomyopathies and/or arrhythmias (Fatima et al. 2011; Schaaf et al. 2011; Itzhaki et al. 2012; Nakamura et al. 2013).

Central to the paradigm that in vitro ECTs are useful as myocardial surrogates is the requirement that the immature cells within these tissues develop into a mature and functional cardiac syncytium. However, ECTs display variable rates of cell survival, proliferation, and maturation depending on cellular composition (Tobita et al. 2006; Stevens et al. 2009; Fujimoto et al. 2011; Kurazumi et al. 2011; Tiburcy et al. 2011; Sekiya et al. 2013), stage of cellular differentiation (Zimmermann et al. 2000; Tobita et al. 2006; Fujimoto et al. 2011; de Lange et al. 2013), matrix and growth factor composition (Tulloch et al. 2001; Ott et al. 2008; Rubbens et al. 2009; Karikkineth and Zimmermann 2013), 3D architecture, and in vitro conditioning (Shimizu et al. 2006; Zimmermann et al. 2006; Fujimoto et al. 2011). It is remarkable that despite this broad range of design features, all ECT related in vitro conditioning protocols to date have shown that immature cardiomyocytes (CM) within ECT positively respond to preconditioning with increased cellular proliferation, hypertrophy, and maturation.

In order to better define the complexity of the in vitro molecular and cellular ECT response to mechanical and/or biochemical conditioning we previously tested the narrow hypothesis that the increase in embryonic chick ECT CM proliferation following cyclic mechanical stretch is associated with increased p38 mitogen-activated protein kinase (p38MAPK) phosphorylation (Clause et al. 2009), similar to the mature cardiomyocyte response to mechanical loading (Sadoshima and Izumo 1993; Seko et al. 1999; Han and Molkentin 2000; Liang and Molkentin 2003; Engel et al. 2004; Tenhunen et al. 2004; Wu et al. 2004; Rose et al. 2010; van Berlo et al. 2013). This finding is consistent with other reports that p38MAPK is involved in the cellular response to mechanical conditioning. Our previous analysis used standard western blot and enzyme-linked immunosorbent assays relevant to p38MAPK and Akt pathways and used a commercial protein kinase inhibitor, SB202190, which inhibits p38MAPKα and p38MAPKβ but not p38 MAPKγ (Kuma et al. 2005). BIRB796 inhibits all p38 MAPK isoforms in vivo and in vitro (Kuma et al. 2005) so we chose this broader p38MAPK inhibitor for the current study. We recognize that p38MAPK is known to affect multiple regulatory pathways and to have differential effects on cardiomyocytes and noncardiomyocytes in vitro and in vivo (Liang and Molkentin 2003; Engel et al. 2004; Xu et al. 2005; Aouadi et al. 2006; Lal et al. 2008; Li et al. 2011). Several additional studies have confirmed the cellular, but not the molecular ECT responses to mechanical loading conditions (Tulloch et al. 2001; Rubbens et al. 2009; Boudou et al. 2012). Therefore, in this study we performed an unbiased investigation of changes in transcript expression in ECTs generated from embryonic rat heart cells now used for cardiac repair and regeneration protocols to test the hypothesis that global ECT gene expression patterns are sensitive to mechanical loading conditions and to tyrosine kinase inhibitors, similar to the maturing myocardium.

## Material and Methods

### Experimental animals

Gestational day 14.5 Sprague Dawley rats (Harlan, Indianapolis, IN) embryos were used to develop an engineered early embryonic cardiac tissue (ECT). Rats were maintained within the animal facility of the Animal Facility, University of Louisville. All experimental protocols followed the National Institutes of Health guidelines for animal care and were approved by the University of Louisville Animal Care and Use Committee.

### Construction of rat ECT

For ECT construction, pregnant mothers were anesthetized using 3% Isoflurane inhalation with 100% oxygen followed by hysterectomy to harvest the uterus and embryos. Immediately after hysterectomy, the mother was euthanized by induced asystole under 5% Isoflurane anesthesia. The excised uteri were transferred to a sterilized petri dish filled with cold (25°C) phosphate buffered saline and 1% antibiotic–antimycotic solution containing 100 units/mL of penicillin, 100 units/mL of streptomycin, and 0.25 mg/mL of amphotericin-B (Invitrogen, Carlsbad, CA), the fetuses were excised by hysterotomy, and then fetal hearts were harvested. The great vessels and atrium were removed from each fetal heart, and ventricular tissue was collected and pooled. Pooled fetal ventricles were then enzymatically digested by 3 mg/mL of collagenase type II followed by 0.05% trypsin-ethylenediaminetetraacetic acid solution (Invitrogen). Isolated cells were preplated for 1 h and then cultured on a gyratory shaker (50 rotations/min) for 16 h to reaggregate viable CMs. Approximately 45k clusters of cardiac were mixed with acid-soluble rat tail collagen type I (Sigma, St Louis, MO) and matrix factors (Matrigel; BD Science, San Jose, CA). Cell/matrix mixture was performed as follows: (1) Cells were suspended within a culture medium (high glucose-modified Dulbecco's essential medium; Invitrogen) containing 20% fetal bovine serum (Invitrogen). (2) Acid-soluble collagen type I solution (pH 3) was neutralized with alkali buffer (0.2 mol/L NaHCO_3_, 0.2 mol/L HEPES, and 0.1 mol/L NaOH) on ice. (3) Matrigel (15% of total volume; BD Sciences) was added to the neutralized collagen solution. (4) Cell suspension and matrix solution were mixed. The final concentration of collagen type I was 0.67 mg/mL.

Cylindrical ECT was constructed using a collagen type I-coated silicone membrane culture plate (Bioflex culture plate; Flexcell International, Hillsborough, NC) and FX-4000TT system (Flexcell International). Briefly, the center of the silicone membrane of a Tissue Train culture plate was deformed by vacuum pressure to form a 20-mm-length × 2-mm-width trough using a cylindrical loading post. Approximately 200 μL of cell/matrix mixture, containing ∼1 × 10^6^ cells, was poured into the trough and incubated for 150 min in a standard CO_2_ incubator (37°C, 5% CO_2_) to form a cylindrical construct. Both ends of the construct were held by anchors attached to the Tissue Train culture plate. When the tissue was formed, the culture plate was filled with a growth medium containing 10% fetal bovine serum (Invitrogen), 1% antibiotic–antimycotic solution (Atlanta Biologicals, Norcross, GA), and 1% chicken embryo extract. Constructed engineered tissues were cultured for 7 days and the culture medium was changed every other day (Fujimoto et al. 2011).

### Mechanical stretch stimulation and p38MAPK inhibition

To determine the effect of cyclic mechanical stretch and/or p38MAPK inhibition on ECT gene changes, we exposed culture day 5 ECT to uniaxial cyclic mechanical stretch (0.5 Hz and 5% elongation), to p38MAPK inhibition by BIRB796 (Catalog No. S1574, Selleck Chemicals, Houston, TX, 10 μmol/L), or both stretch and BIRB796 (Anur et al. 2012; Korhonen et al. 2012). We have previously noted that 0.5 Hz and 5% strain of cyclic stretch increased both ECT contractile force generation and cellular proliferation activity with minimal technical ECT loss due to detachment of the tissue from anchors of the culture plate (Clause et al. 2009). We performed cyclic mechanical stretch stimulation or p38MAPK inhibition for 48 h beginning on culture day 5. For p38MAPK inhibition, we treated ECTs with BIRB796-containing culture medium.

### Isolation of RNA from ECT

We generated individual ECT for each experimental group (control *n* = 7, stretch *n* = 7, BIRB *n* = 4, and stretch+BIRB *n* = 4). RNA was isolated and DNA was generated from individual ECT as biologic replicates, not technical replicates from the same ECT. Fresh ECT samples were homogenized by an Omnitip Tissue homogenizer (Cat. No.: 6615-7273, USA Scientific, Ocala, FL). Total RNAs were isolated using Invitrogen Trizol and purified by RNeasy® Mini Kit (Qiagen, Valencia, CA; Cat. No.: 74104). Then the RNA quality and quantity were measured using the NanoDrop ND-1000 (Thermo Fisher Scientific Inc., Waltham, MA) and the Bioanalyzer 2100 (Agilent Technologies Inc., Santa Clara, CA).

### DNA microarray and hybridization

High-quality RNA samples were processed for genome-wide transcript expression using Agilent Rat GE 4 × 44K V3 microarrays (Agilent Technologies Inc.). The Low Input Quick Amp Labeling Kit (Agilent Technologies Inc.) was used for labeling and hybridization. The samples of total RNA were labeled with the aid of T7 RNA polymerase in the presence of Cy3-CTP. The Cy3-labeled RNAs were purified using RNeasy MiniElute Cleanup kit (Qiagen). The yield and label incorporation efficiencies were measured with the aid of a spectrophotometer (NanoDrop Lite, Thermo Fisher Scientific, Inc). Each labeled cRNA of 1.65 μg was fragmented at 60°C for 30 min (Agilent Gene Expression Hybridization Kit) and then hybridized to rat whole genome 4 × 44K oligo microarray v3 slide (Agilent Technologies Inc.) at 65°C for 17 h. The slides were washed with 0.005% Triton-X100/Wash Buffer I (Agilent Technologies Inc.) at room temperature for 1 min, and by 0.005% Triton-X100/Wash Buffer II at 37°C for 1 min.

### Image acquisition and quantification

The slides were scanned with the aid of the Agilent DNA microarray scanner G2505C (Agilent Technologies Inc.). The one-color microarray images (*.tif) were extracted with the aid of Feature Extraction 11.0 (Agilent Technologies Inc.). The raw data files (*.txt) were imported into GeneSpring (GX 11.1), normalized, and analyzed. Transcript data were uploaded to the Gene Expression Omnibus NCBI public functional genomics data repository prior to manuscript review.

### Reverse transcription and quantitative real-time PCR

Selected genes identified from the microarray analysis were chosen for validation of changes in transcript expression by quantitative real-time PCR (qPCR). These secondary analyses used the same RNA samples that were previously used for the microarray. qPCR was performed in triplicate using the TaqMan® Gene Expression Master Mix and the ABI 7900HT (Applied Biosystems, Foster City, CA). Data were normalized to ribosomal protein L13a (RPL13a). Total RNA of 2 μg for each of the samples was used for the RT reaction to generate cDNA with the aid of the High Capacity cDNA Reverse Transcription Kit (Applied Biosystems) and TaqMan® Gene Expression Assays (Applied Biosystems), including gene-specific primer pairs (Table 1).

**Table 1:** Gene-specific expression assays primer pairs

Assay ID	Gene symbol	Gene
Rn01413923	EREG	Epiregulin
Rn01407421	p38 MAPKγ	MAP kinase 12
Rn01332449	MYH3	Myosin heavy chain 3
Rn01520922	MAP3K6	MAP kinase kinase 6
Rn01503668	MYBPC1	Myosin-binding protein C
Rn00567471	AREG	Amphiregulin
Rn00695128	FGF8	Fibroblast growth factor 8
Rn00564119	FOSL1	FOS-like antigen 1
Rn01425264	REG3a	Regenerating islet-derived 3 alpha
Rn01640664	IL1R1L	Interleukin 1 receptor-like 1
Rn01483828	PTGS2	Prostaglandin-endoperoxide synthase 2
Rn00821946	RPL13a	Ribosomal protein L13a
Rn01527840	Hprt1	Hypoxanthine P-ribosyltransferase 1

### Statistical analysis

These microarray experiments were completed using multiple Agilent chips. We normalized the gene transcript expression values for the control samples on each chip and then compared differentially expressed mRNAs between control and either Stretch, BIRB, or Stretch+BIRB groups using unpaired Student's *t*-test with significance assigned to *P*-values ≤0.05 and/or fold change values ≥1.5. Because data were normalized from several individual chips we chose isolated Student's *t*-tests rather than analyses of variance (ANOVA) between all four groups. Biological functions and pathways, for these differentially expressed genes were determined with the aid of ingenuity pathway analysis (IPA) software (Ingenuity Systems, Redwood City, CA). Heat maps were generated using Partek Genomics Suite software (version 6.6, Partek Incorporated, Life Technologies, Carlsbad, CA). Data were also analyzed using MetaCore software (Thomson Reuters, New York, NY) to identify pathways enrichment for the three treatment groups. Statistical significant differences in expressed mRNAs identified with the aid of GeneSpring software (GX 11.1) were determined by unpaired Student's *t*-test with a cutoff *P*-value of ≤0.05 and fold change of ≥1.5 (Caldwell et al. 2010; Dalman et al. 2012; Maleki et al. 2013).

## Results

### Transcript expression fold changes

**Figure 1:**
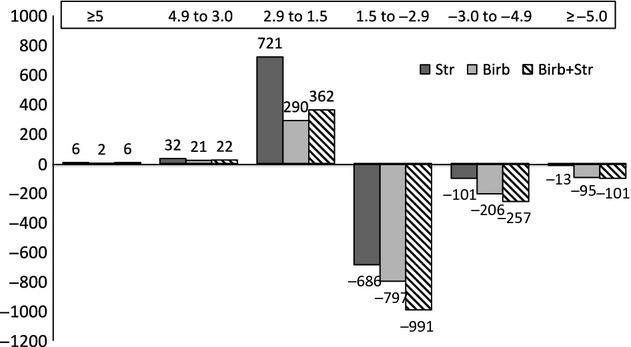
Engineered cardiac tissue transcript expression changes at least 1.5-fold measured by microarray in response to stretch (dark solid bar), BIRB796 (gray solid bar), or stretch+BIRB796 (dashed bar). Note that most transcripts increased by less than threefold (above the *X*-axis) or decreased by less than fivefold (below the *X*-axis).

**Figure 2:**
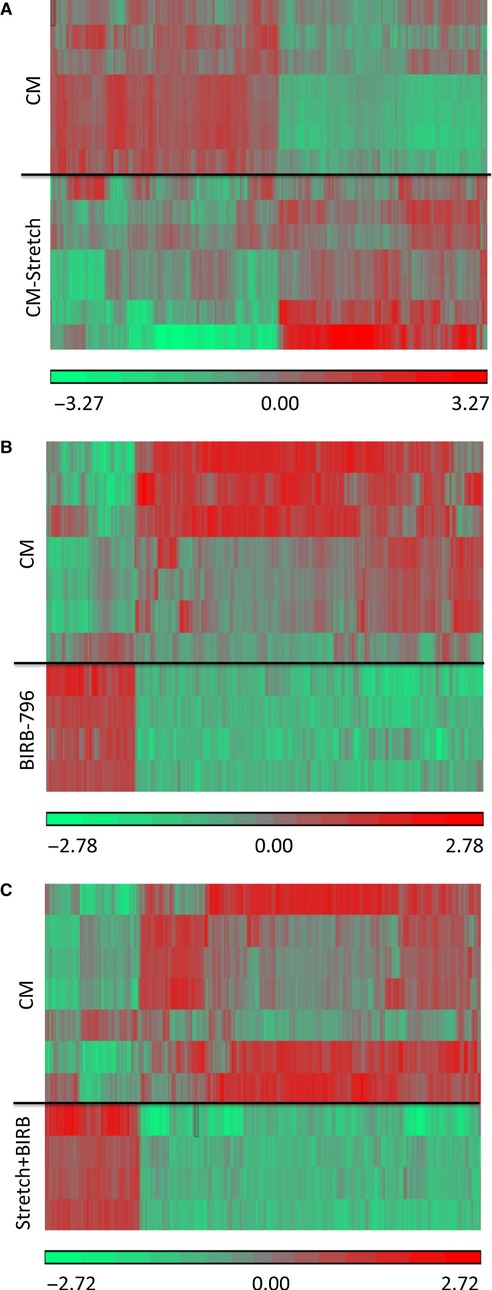
Hierarchical clustering of differentially expressed transcripts in engineered cardiac tissues. Each row represents an individual sample and each band represents a single transcript (control *n* = 7, stretch *n* = 7, BIRB *n* = 4, and stretch+BIRB *n* = 4). The change in gene expression is represented by the color range. (A) Control specimens compared to specimens exposed to 48 h of cyclic stretch. (B) Control specimens compared to specimens exposed to 48 h of BIRB796. (C) Control specimens compared to specimens exposed to 48 h of cyclic stretch and BIRB796. (*P* ≤ 0.05, fold change ≥1.5, normalized intensity).

Our initial analysis of the changes in ECT global transcript expression in response to 48 h of cyclic mechanical stretch, p38MAPK inhibition with BIRB796, or combined stretch and BIRB796 revealed a large number of altered transcripts (Table 2). As would be expected for a complex, multicellular biologic tissue, a large number of transcripts were altered by mechanical stretch and a greater number of transcripts were altered by the “selective” BIRB796 inhibitor or by combined treatment. Figure 1 displays the number of transcripts either increased or decreased by these three conditions. The majority of altered transcripts were increased by less than threefold or decreased less than fivefold compared to expression levels in control ECTs. Comparison changes in transcript expression are also displayed in the hierarchical expression maps (Fig. 2). Figure 2A shows the transcript expression from seven control ECT samples and from seven samples derived from ECTs after 48 h of mechanical stretch. It is worth noting that there appear to be differences between the specimens within groups that may relate to variability in cellular content. Figure 2B shows the transcript expression from seven control ECT samples and from four samples derived from ECTs after 48 h of tyrosine kinase inhibition with BIRB796. Figure 2C shows the transcript expression from seven control ECT samples and from four samples derived from ECTs after the combination of 48 h of cyclic mechanical stretch and tyrosine kinase inhibition with BIRB796. Note that both groups treated with BIRB796 had very distinct heat maps in comparison to controls.

**Table 2:** Transcript responses to stretch, BIRB796, or stretch + BIRB

Treatment group	Total IDs	Mapped IDs	Unmapped IDs
Stretch	3704	3245	459
BIRB796	4460	3907	553
Stretch + BIRB796	5527	4856	671

*P* < 0.05 determined by Ingenuity Pathway Analysis.

**Figure 3:**
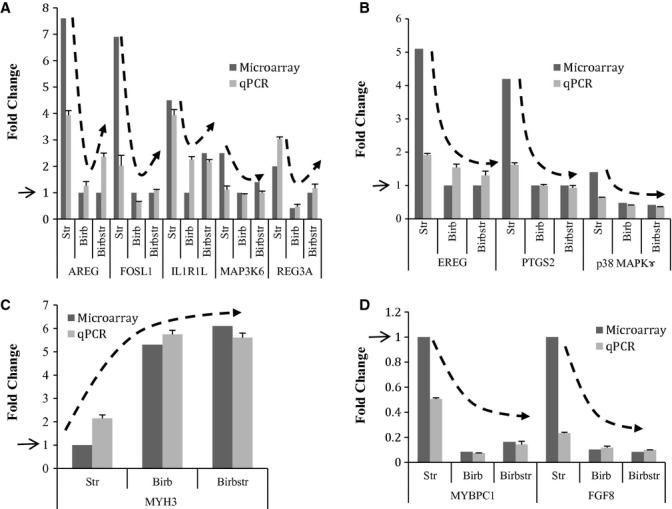
Changes in selected engineered ECT expression measured by microarray and q-PCR in response to mechanical stretch conditioning and/or p38MAPKinase inhibition with BIRB796 (control *n* = 7, stretch *n* = 7, BIRB *n* = 4, and stretch+BIRB *n* = 4). (A) Transcripts noted to increase in response to Stretch, decrease in response to BIRB796, and then increase with the combination of Stretch+Birb796. (B) Transcripts noted to increase in response to Stretch, decrease in response to BIRB796, and not increase with the combination of Stretch+Birb796. (C) Transcripts noted to increase in response to Stretch and then further increase in response to BIRB796 or the combination of Stretch+Birb796. (D) Transcripts noted to decrease in response to Stretch and then further decrease in response to BIRB796 or the combination of Stretch+Birb796. Arrow along the *Y*-axis refers to control transcript expression.

We then determined the transcripts with the greatest increase in expression (Table 3) or decrease in expression (Table 4) for validation using quantitative PCR. These transcripts represent a wide range of regulatory pathways beyond our initial hypothesis related to the role of p38MAPK in the adaptive role of ECT to loading. Figure 3 provides a comparison between fold changes in transcript expression by microarray versus qPCR. For some of the transcripts we noted an increase in expression in response to mechanical stretch that was reduced by p38MAPK inhibition by BIRB796 and then partially reversed by concurrent stretch and BIRB796 treatment (Fig. 3A). For other transcripts a similar response to stretch and to BIRB796 was also noted, but without a reversal in the BIRB796 effect by stretch (Fig. 3B). For myosin heavy chain 3 (MYH3), only qPCR showed an increase in transcript expression following BIRB796 treatment (Fig. 3C). Concurrent stretch and BIRB796 treatment increased transcript expression similar to BIRB796 treatment alone. Finally we noted a decrease in transcript expression for myosin-binding protein C (MYBPC1) and fibroblast growth factor 8 (FGF8) in response to stretch for qPCR only and then a further decrease in response to either BIRB796 or BIRB796 and stretch (Fig. 3D).

**Table 3:** Transcripts with the largest increase in expression (top 10)

Stretch (fold)		BIRB (fold)		Stretch + BIRB (fold)	
CHDR1	8.2	GRM8	9.0	STMN4	7.1
AREG*	7.6	MYH3*	5.3	MYH3*	6.1
FOSL1*	6.9	RGS4	4.7	RGS4	5.3
GNAT2	5.2	ADH7	4.3	ACTG2	4.8
EREG*	5.1	STMN2	4.3	GLDN	4.6
PKP1	4.9	LMOD1	4.2	GRM8	4.4
IL1R1L*	4.5	AK5	4.0	CRLF1	4.3
PTGS2*	4.3	OR51E2	3.8	STMN2	4.1
CALCA	4.1	MYH11	3.7	CALD1	4.0
SRXN1	4.0	ACTG2	3.6	LMOD1	4.0

* Indicates transcripts selected for qPCR validation.

**Table 4:** Transcripts with the largest decrease in expression (top 10)

Stretch (fold)		BIRB (fold)		Stretch + BIRB (fold)	
SLC6A1	−8.0	HPD	−17.0	HPD	−21.9
LHX8	−7.1	POU4F4	−12.3	POU4F4	−16.6
PLAC8	−6.9	CYSS	−12.1	MMP7	−15.0
SLITRK6	−6.3	CEACAM1	−11.9	FGF8*	−11.9
AQP4	−6.2	MYBPC1*	−11.7	CYSS	−10.9
IGFBP5	−5.6	FGF8*	−9.8	RETN	−9.6
SLC9A9	−5.3	OTOG	−9.7	C16ORF89	−9.4
USP17L2	−5.2	KCNK1	−9.7	SLAMF6	−9.1
MPZL2	−5.1	PON1	−9.1	MMP9	−8.4
KCNH2	−5.1	SPOCK2	−7.8	F13A1	−8.1

* Indicates transcripts selected for qPCR validation.

### Regulatory network responses

**Figure 4:**
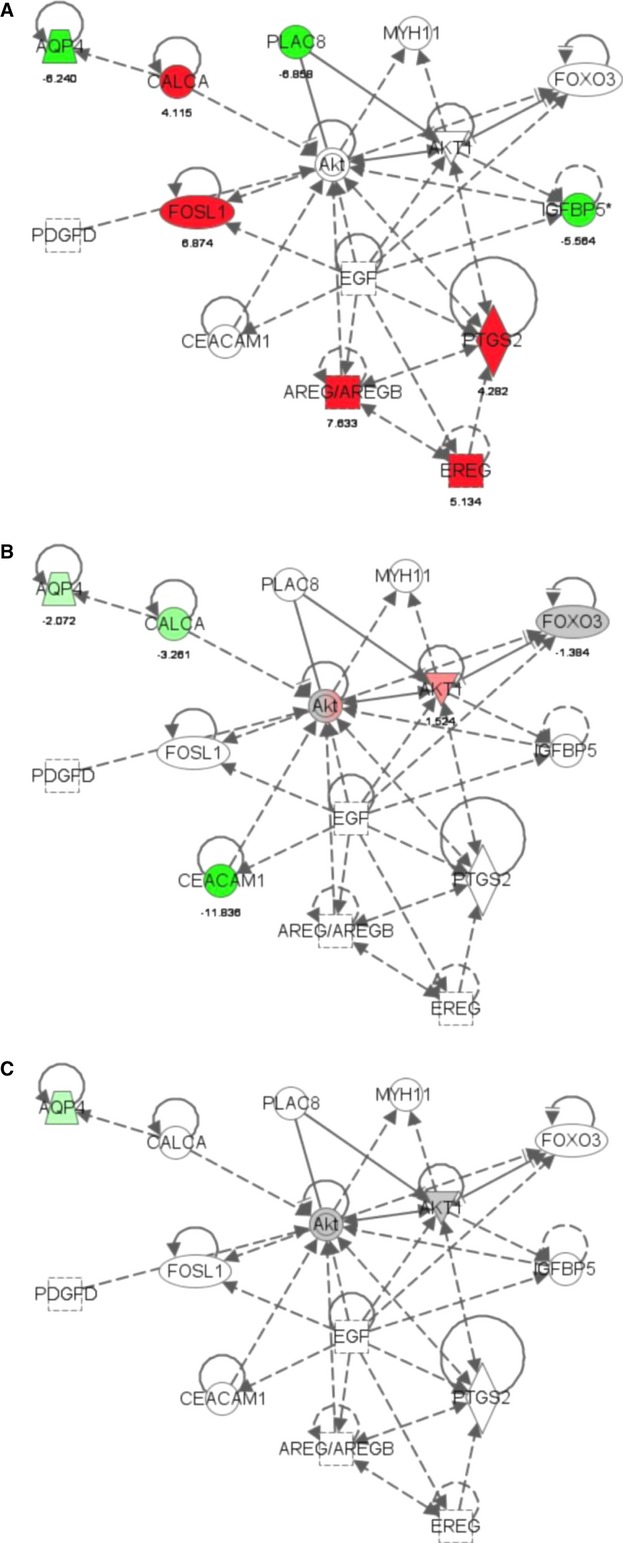
Representative ingenuity pathway analysis (IPA) of relationship between significant genes involved in the response to mechanical stretch and/or BIRB796 treatment in engineered cardiac tissues. IPA biological/gene network analysis indicates the interactions between groups of genes and how expression decreases (in green) or increases (in red). (A) Transcripts after 48 h of cyclic stretch. (B) Transcripts after to 48 h of BIRB796. (C) Transcripts after 48 h of cyclic stretch and BIRB796. These transcripts appear to interact via the Akt complex and AKT1 kinase.

As would be expected for an adaptive response, the experimental groups showed significant changes in regulatory networks related to cellular growth and proliferation, development, movement, cell signaling, and cardiovascular development and function. Table 5 displays five representative regulatory networks with between 214 and 437 altered transcripts within those networks depending on the experimental group (IPA software). We used the IPA software to identify associations between the top 10 molecules altered in the three groups (Fig. 4). These molecules appear to interact via the Akt complex and AKT1 kinase, though the absolute value for AKT1 transcript level was only altered in the BIRB796 treatment group. Table 6 shows an expanded list of pathways enriched in response to the three treatment groups and ranked by *P*-value (MetaCore software). Most of the pathways involve cytoskeletal remodeling, development, cell adhesion, chemotaxis, and immune responses though rank order varied between the groups. Surprisingly, only a small number of the most significantly altered pathways included p38MAP kinase-related transcripts. Thus, the response to cyclic mechanical loading involved a diverse adaptive response involving multiple pathways and the transcript response to BIRB796 treatment suggests a biologic effect much broader than the p38MAP kinase pathways.

**Table 5:** Number of altered transcripts for top regulatory functions

Regulatory network	Stretch	BIRB	Stretch + BIRB
Cellular growth and proliferation	437	354	436
Cellular development	395	336	418
Cellular movement	300	262	320
Cardiovascular devel. and function	221	215	230
Cell-to-cell signaling and interaction	214	308	361

**Table 6:** Regulatory pathways altered by stretch, BIRB796 or combined treatment

Pathway enrichment by stretch	
Cytoskeleton remodeling	TGF, WNT, and cytoskeletal remodeling*
Development	Regulation of epithelial-to-mesenchymal transition
Development	WNT signaling pathway Part 2
Cytoskeleton remodeling	Neurofilaments
Immune response	IL-1 signaling pathway*
Cytoskeleton remodeling	Cytoskeleton remodeling*
Development	Hedgehog signaling
Cytoskeleton remodeling	Regulation of actin cytoskeleton by Rho GTPases
Cytoskeleton remodeling	Reverse signaling by ephrin B
Normal and pathological	TGF-beta-mediated regulation of cell proliferation*
Pathway enrichment by BIRB796	
Development	WNT signaling pathway Part 2
Cell adhesion	Cell matrix glycoconjugates
Cytoskeleton remodeling	TGF, WNT, and cytoskeletal remodeling*
Development	Notch signaling pathway
Development	Regulation of epithelial-to-mesenchymal transition
Cell adhesion	Ephrin signaling
Cell adhesion	Tight junctions
Development	A2B receptor: action via G-protein alpha s*
Cell adhesion	Chemokines and adhesion
Chemotaxis	Leukocyte chemotaxis
Pathway enrichment by stretch + BIRB796	
Development	Regulation of epithelial-to-mesenchymal transition
Chemotaxis	Leukocyte chemotaxis
Cell adhesion	Cell matrix glycoconjugates
Immune response	Role of HMGB1 in dendritic cell maturation and migration*
Cytoskeleton remodeling	Keratin filaments
Cell adhesion	Plasmin signaling*
Cell adhesion	Chemokines and adhesion
Immune response	Immunological synapse formation
Cell adhesion	Tight junctions
Cell adhesion	Ephrin signaling

* *P* < 0.05 change in p38 MAP kinase pathway-related transcripts.

## Discussion

This study provides the first description of the changes in global in vitro ECT gene transcript expression profile in response to 48 h of mechanical loading and/or p38MAPK inhibition with BIRB796. The major findings are (1) ECT mechanical loading and/or p38MAPK inhibition alters the expression of a large number of transcripts and regulatory networks and/or pathways, as would be expected by an adaptive biologic response; (2) most of the altered transcripts induced by BIRB796 were outside of the p38MAPK pathways; and (3) for many transcripts the responses to mechancial loading and to BIRB796 inhibition were opposite and concurrent treatment with stretch partially reversed the BIRB796 effect for some but not all transcripts.

### Altered transcript expression and regulatory networks in response to mechanical loading with or without BIRB796 treatment

The primary goal of the current study was to better define the changes in transcript expression that occur within ECT in response to the epigenetic stimulation produced by cyclic mechanical stretch (Kurazumi et al. 2011; Maleki et al. 2013). Previous experiments have shown that immature CM within ECT positively respond to mechanical loading with increased cell proliferation and functional maturation with the suggestion that p38MAPK pathways are involved in this adaptive response. We found a large number of altered transcripts following mechanical stretch and to our surprise we noted the BIRB796 effect was much broader than would be expected from this “selective” p38MAP kinase inhibitor. We noted variations in transcript expression within experimental groups that likely represents biologic variation in cellular composition. We selected several transcripts for validation by qPCR based on their fold change in expression or relevance to CM, primarily when there appeared to be differential effects between mechanical stretch and BIRB796 treatment.

#### Amphiregulin and epiregulin

We noted a significant increase in transcript expression for Amphiregulin (AREG) and Epiregulin (EREG) in response to 48 h of mechanical stretch. AREG and EREG are both members of the epidermal growth factor (EGF) family, function as autocrine growth factors, and have been described to be involved in the response to mechanical loading in mesodermal-derived tissues including cardiomyocytes, fibroblasts, and vascular smooth muscle (Deacon and Knox 2010; Marshall et al. 2010; Shao and Sheng 2010). EGF receptor activation via recombinant EGF has been shown to stimulate proliferation over differentiation in cultured human fetal ventricular myocytes (Goldman et al. 1996). It is worth noting changes in AREG protein excretion in response to G protein-coupled receptor agonists such as Endothelin-1 (ET-1), which function as in vitro surrogates for mechanical stimulation, can occur within the 48 h treatment window of the current experiment (Deacon and Knox 2010; Marshall et al. 2010). This effect is consistent with activation of the RhoA, c-Jun N-terminal kinases (JNKs), and/or p38-MAPKs. The AREG promoter includes transcription factor binding sites relevant to cardiovascular tissues including STAT5A (Foshay et al. 2005; Frias et al. 2007), AP-1 (Goswami et al. 2001; Frias et al. 2007), c-Jun (Nadruz et al. 2005; Onizuka et al. 2012), activating transcription factor-2 (ATF-2) (Monzen et al. 2001), cAMP response element-binding protein (CREB), Husse and Isenberg 2010), and the forkhead box transcription factors (Puthanveetil et al. 2013; Sengupta et al. 2013). EREG can also function as a ligand of most members of the ERBB (v-erb-b2 oncogene homolog) family of tyrosine kinase receptors (Kannan et al. 1997). The EREG promoter includes transcription factor binding sites relevant to cardiovascular tissues including Nkx2.5, HOXA9 (Zaffran and Kelly 2012), and IRF-1 (Kinugawa et al. 1997). It is interesting to note that both AREG and EREG transcript expression decrease in response to BIRB796 treatment, though the mechanism by which MAPK inhibition leads to decreased AREG and EREG transcription is unclear.

#### FOS-like antigen

We also noted a significant increase in transcript expression for FOS-like antigen (FOSL1) in response to mechanical stretch. FOSL1, also known as Fra-1, is a leucine zipper protein that can dimerize with proteins of the JUN family, thereby forming the transcription factor complex AP-1. The FOS proteins have been implicated as regulators of cell proliferation and differentiation of endothelial cells (Evellin et al. 2013), as a negative regulator of αv and β3 integrin expression, a regulator of inflammatory responses, and thus, may have an important role in regulating non-CM proliferation and maturation within ECT. It is also worth noting that FOSL1 belongs within the AP-1 family of dimeric transcription factors noted to regulate AREG transcription. The FOSL1 promoter includes transcription factor binding sites relevant to cardiovascular tissues including the TATA box binding protein, TATA box binding protein (Bhavsar et al. 2000; Angelis et al. 2008), signal transducer and activator of transcription 3 (Wang et al. 2004; Fischer and Hilfiker-Kleiner 2007), p53, and p300 (Schueler et al. 2012). The FOSL1 promoter has multiple ATF-2 binding sites and ATF-2 is a target for SMADS (Kannan et al. 1997) and miRNA-204 (Xiao et al. 2012). Overexpression of ATF-2 increases human cardiomyocyte progenitor proliferation and differentiation (Xiao et al. 2012). ATF-2 overexpression promoted human cardiac progenitor cell proliferation, further demonstrating the role played by ATF-2 as a target gene of miRNA-204. Therefore, miRNA-204 is required for human cardiomyocyte progenitor cell differentiation and ATF-2 is a target gene of miRNA-204 in human cardiac progenitor cells. This study indicates that miRNA-204 is among the regulators that drive progenitor cell proliferation and differentiation, and miRNA-204 might be used to influence cell fate.

#### Interleukin-1 receptor-like 1

Mechanical stretch increased the transcript expression of Interleukin-1 receptor-like 1 (IL1RL1), while BIRB 796 treatment suppressed this stretch-mediated increase in IL1RL1 transcription. IL1RL1, also known as protein ST2, has both transmembrane (ST2L) and soluble (sST2) isoforms, and is induced by cardiomyocyte mechanical stretch and injury (Weinberg et al. 2002; Sanada et al. 2007). One unique aspect of ST2 is that the agonist for ST2, IL-33, is produced by cardiac fibroblasts as a paracrine regulator of myocardial adaptation to stress and can suppress angiotensin II- and phenylephrine-induced cardiomyocyte hypertrophy (Sanada et al. 2007). Suppression of IL-1 by BIRB796 has been shown to reduce the expression of matrix proteinases in cardiac myofibroblasts, relevant to the remodeling response following cardiac injury (Turner et al. 2010). ST2 has been validated as a biomarker for cardiovascular stress in the Franmingham Heart Study (Wang et al. 2012). In human cardiac tissues, endothelial cells seem to be the source of sST2 and the target for the IL-33 expressed in the nucleus of human adult cardiac fibroblasts and myocytes and released during necrosis (Demyanets et al. 2013). The ILIRL1 promoter contains multiple cardiac-relevant transcription factors binding sites including MEF-2A (Ewen et al. 2011) and Yin Yang 1 (YY1) noted to be involved in fetal cardiomyocte maturation (Nan and Huang 2009) and cardiac mesodermal differentiation from cardiac progenitor cells (Gregoire et al. 2013). Of note the FOSL1 promoter also has multiple binding sites for YY1.

#### Cyclooxygenase 2

The Cyclooxygenase 2 (COX2), also referred to as Prostaglandin-Endoperoxide Synthase 2 (PTGS2), also increased in response to stretch and then decreased in response to BIRB796. COX2 is the key enzyme in prostaglandin biosynthesis and acts both as a dioxygenase and as a peroxidase. There are two isozymes of PTGS: a constitutive PTGS1 and an inducible PTGS2, which differ in their regulation of expression and tissue distribution. COX2 is regulated by specific stimulatory events, suggesting that it is responsible for the prostanoid biosynthesis involved in proliferation and inflammation. COX2 has been shown to be regulated by p38MAPK in rat CM (Sun et al. 2008). Cardiomyocyte COX2 expression and hypertrophy is also enhanced by Heparin-binding EGF-like growth factor stimulation via the MEK5-ERK5 signaling cascade and MEF2 (Lee et al. 2011). As with AREG, COX2 increases early and transiently in the cardiomyocyte transcriptome following Endothelin-1 stimulation of cardiomyocyte hypertrophy (Giraldo et al. 2012) and following interleukin 1β and interleukin 33 stimulation (Barrett et al. 2013). Relevant to the decrease in COX2 transcript expression noted after p38MAPK inhibition with BIRB796 in the current study, inhibition of the MAPK-activated protein kinase 2 (MK2) decreases COX2 protein synthesis without affecting mRNA levels (Streicher et al. 2010). Similar to the other transcripts that increased after stretch and then decreased by BIRB, the COX2 promoter contains multiple cardiac-relevant binding sites including STAT5A (Yamaura et al. 2003), and ATF-2, also found in the IL1RL1 promoter. The COX2 promoter also contains a binding site for the peroxisome proliferator-activated receptor (PPAR-alpha) binding sites known to regulate cardiomyocyte length and hypertrophy (Duan et al. 2005; Liang et al. 2010; Hinrichs et al. 2011).

#### Regenerating islet-derived 3 alpha (REG3A)

We noted an increase in regenerating islet-derived 3 alpha (REG3A), a secretory stress protein usually involved in the pancreatic response to stress and injury, in response to mechanical stretch that decreased following BIRB796 treatment and then partially reversed by concomitant BIRB796 and stretch. This transcript has not been described in cardiac tissues, however, the promoter region includes regulatory elements for the sterol regulatory element binding protein, SREBP1a-1c, shown to be regulated in response to cardiomyocyte stress (Park et al. 2008), and Evi-1, which regulated calreticulin in the embryonic heart (Qiu et al. 2008).

#### Fibroblast growth factor 8

One interesting change in gene expression occurred with Fibroblast Growth Factor 8 (FGF8), known to regulate early cardiac morphogenesis, specifically related to the expansion of the embryonic cardiac outflow tract from anterior heart field-derived mesoderm (Park et al. 2006) with a potential role of FGF8 as a chemotactic factor for migrating neural crest cells (Sato et al. 2011). Of note, in addition to FGF2 (House et al. 2010), FGF8 has been found to regulate cardiac size and proportion during development (Marques et al. 2008). The FGF8 promoter includes binding sites for Nkx2.5, serum response factor (SRF), MEF2A, as well as AP-1, multiple Forkhead box O transcription factor (FoxO) and GATA1-3 factors, and many other cardiac-relevant transcription factors. FGF may act via intracellular P38 MAPK, ERK1/2, and CREB signaling during early heart development (Keren-Politansky et al. 2009; Woznica et al. 2012), suggesting one explanation for the decrease in FGF8 expression following BIRB796 treatment.

#### Myosin-binding protein C, slow type (MYBPC1)

We noted a significant decrease in MYBPC1 expression following BIRB796 treatment that was partially reversed by mechanical stretch. Myosin-binding protein C (MYBPC1) family members are myosin-associated proteins found in the cross-bridge–bearing zone (C region) of A bands in striated muscle. The MYPBC1 protein is the slow skeletal muscle isoform of MYBPC1 and plays an important role in muscle contraction by recruiting muscle-type creatine kinase to myosin filaments. In vitro MYBPC1 binds myosin heavy chain, F-actin, and native thin filaments, and modifies the activity of actin-activated myosin ATPase. MYBPC mutations have been associated with human cardiomyopathies (Maron et al. 2001; Knöll 2012) and MYBPC activation requires phosphorylation by phosphokinase A which can be influenced by p38 MAPK inhibition in cardiac fibroblasts (Husse and Isenberg 2010). The MYBPC1 promoter contains multiple cardiac-relevant regulatory binding sequences including MEF2 and MEF2A, Nkx2.5, SRF, STAT5A, and STAT5B.

#### Myosin heavy chain 3, embryonic skeletal muscle (MYH3)

In contrast to the decrease in MYBPC1 expression following BIRB796 treatment, we noted an increase in MYH3 transcript expression in response to BIRB796 that was not influenced by concomittant mechanical stretch. MYH3 is a member of the MYH family and encodes a protein with an IQ domain and a myosin head-like domain (Eller et al. 1989). MYH3 is usually described in embryonic skeletal muscle development, though the transcript and protein have been noted in the embryonic heart (Rutland et al. 2011; Wang et al. 2013) as have been other skeletal muscle myosin isoforms (Clause et al. 2012). Similar to the MYBPC1 promoter, the MH3 promoter contains multiple cardiac-relevant regulatory binding sequences including AP1, CREB, HOXA3, Nkx2.5, STAT5A, YY1, c-Fos, c-Jun, and c-Myb. The cAMP response element binding protein, CREB, is known to regulate cardiac myocyte proliferation (Goldspink and Russell 1994) in part via p38 MAPK (Markou et al. 2004) and Akt (Shanmugam et al. 2011).

#### Altered p-38 mitogen-activated protein kinase (p38 MAPKγ or MAPK12)

The original premise for these experiments was to test the hypothesis that the molecular response to mechanical loading in an ECT derived from immature CM was transduced, in part, via p38 MAPK-related pathways and would be altered by p38 MAPK inhibition by BIRB796. To our surprise, we noted that very few members of the p38 MAPK pathway had alterations in transcript expression beyond 1.5-fold. We noted only a modest increase in p38 MAPKγ transcript expression in response to 48 h of mechanical stretch, though transcript levels were clearly reduced in response to BIRB796 treatment. The protein encoded by p38 MAPKγ has been identified in neonatal rat CM as well as in fast, slow, and mixed skeletal muscle (Court et al. 2002; Braz et al. 2003). p38 MAPKγ acts as a signal transducer during the differentiation of myoblasts into myotubes and is normally active in slow skeletal muscle (Foster et al. 2012). This finding complements our previous observation in embryonic chick cardiomyocyte-derived ECTs where we noted an increase in p38 MAPK phosphorylation but not an increase in protein content as measured by a monoclonal antibody for p38 MAPKα (Clause et al. 2009). In that study we also noted that p38 MAPK inhibition with SB202190 reduced cardiomyocyte proliferation and active stress production by ECT (Clause et al. 2009). However, that study did not explore changes in the transcript or protein levels for other MAPK family members or regulatory kinases. It is important to note that p38 MAPK inhibitors can be selective, as SB203580 inhibits p38MAPKα and p38MAPKβ but not p38 MAPKγ (Kuma et al. 2005). BIRB796 inhibits all p38 MAPK isoforms in vivo and in vitro (Kuma et al. 2005). As with the other genes noted to have altered transcript expression in response to ECT mechanical strecth, the p38 MAPKγ promotor includes binding sites for cardiac transcription factors inlcuding CREB, NF-kappaB, AP-1, CREB, Nkx2.5, SRF, STAT2-6, etc.

#### Altered mitogen-activated protein kinase related kinase (MAP3K6)

We did find a significant increase in MAP3K6 expression in response to mechanical stretch and a decrease in response to BIRB796 treatment. Interestingly, MAPK Kinase Kinase (MAP3K6) is serine/threonine protein kinase and has been shown to activate JNK but not ERK or p38 kinase pathways. MAP3K6 has been shown to regulate vascular endothelial growth factor expression in vascular endothelial cells (Eto et al. 2009), however, the role of MAP3K6 in cardiac tissues remains unknown. Data were not available on the rat MAP3K6 promotor, however, the mouse MAP3K6 promoter includes binding sites for many of the same cardiac transcription factors inlcuding AP-1, CREB, HOXA3, SRF, STAT5A, YY1, etc.

#### Summary of shared transcription factors

Table 7 includes a summary of some of the transcription factors shared between genes found to be up- or downregulated in response to ECT mechanical stretch and/or BIRB796 inhibition. Most of these transcription factors have been described in cardiac cells or tissues though there are some binding sites that appear to be novel, for example FOXD1, POU2F1, SREBP-1a-c. Clearly there are many common factors and we do not know which of these regulatory elements are responsible for triggering or modifying these adaptive responses.

**Table 7:** Transcription factor binding sites (and number per gene) for selected genes noted to alter transcription in response to stretch, BIRB796, or stretch + BIRB796

TF binding sites	AREG	EREG	IL1RL1	PTGS2	MYH3	MYBPC1	MAPK3K6	FOSL1	MAPK12	FGF8	REG3A
*AP-1* (Lal et al. 2008; Lee et al. 2011)	3	3	1		2		2	5	2	2	
*AREB6*	1			1	1	1	4	1	2	3	1
*ATF-2* (Monzen et al. 2001; Li et al. 2011)	1			1				2	1		
*cdc5*	1	1	1	1							
*C-Jun* (Lal et al. 2008; Lesman et al. 2010)	2	1			2		2	5	1	7	
*CREB* (Li et al. 1999; Stevens et al. 2009; Streicher et al. 2010; Turner et al. 2010)	1				2		3	5	7	6	
*Evi-1* (Sengupta et al. 2013)				2	1	1		1	2	2	1
*FOXD1* (Liang and Molkentin 2003; Liang et al. 2010)	2	1		2		1					
*FOXJ2* (Liang and Molkentin 2003; Liang et al. 2010)	1	1	1	2			1			4	
*GATA-1*			2		2	2	3	6	2	6	
*GATA-2*			1				1	1		1	
*GATA-3*			2				1	1		1	
*HOXA9* (Markou et al. 2004)		1	1					1		3	
*IRF-1* (Maron et al. 2001)		1					1			1	
*MEF-2A* (Park et al. 2008; Rubbens et al. 2009)	2		2			5				6	
*MyoD*			1		1		2	3			
*NF-kB*				1			1	4		2	
*Nkx2.5*	1	2	1		1	2		2	2	1	
*p300* (Nakamura et al. 2013)								2	1	1	
*p53*		1			1	1	3	4	1	4	
*Pax-4a*	1		1	1	2	1	3	4	1	7	
*Pax-6*				1	1	2	2	4	2	2	1
*POU2F1*	3	1		1		3		1	1	7	
*POU3F2*						1			1	2	
*PPAR* (Schaaf et al. 2011; Schueler et al. 2012; Sekiya et al. 2013)				1	1	1	1	5	2	2	
*SEF-1*	1			1			3	1	1		
*SREBP-1a* (Seko et al. 1999)	1	1		2	1		3	1	1	1	1
*SREBP-1b* (Seko et al. 1999)	1	1		2	1		3	1	1	1	1
*SREBP-1c* (Seko et al. 1999)	1	1		2	1		3	1	1	1	1
*SRF*			1	1	1	1	2	2	1	4	
*STAT3* (Monzen et al. 2001; Nadruz et al. 2005)					1					1	
*STAT5A* (Kuma et al. 2005; Kurazumi et al. 2011; Sato et al. 2011)	1			1	2	1	3	1	3	2	
*YY1* (Qiu et al. 2008; Puthanveetil et al. 2013)			1		1		1	5			

### Limitations of experimental design and analyses

Several limitations should be noted with the current study. First, our ECTs are generated using pooled ventricular cells derived from multiple embryos at 14.5 days of gestation. The experimental protocol is designed to generate ECT from cells at a similar point in development though there can be some maturational variation within and between experiments. The process of preplating followed by rotation culture prior to ECT formation results in the selection and incorporation of predominantly viable CM over other cell types. However, these ECTs include both CM and non-CMs with ∼93% CM in day 7 embryonic chick ECT (Clause et al. 2009) and 60% in ED14.5 rat ECT (Fujimoto et al. 2011). The ECT biologic response to our mechanical loading protocol (uniaxial cyclic mechanical stretch, 0.5 Hz, 5% elongation, 48 h) may also have been influenced by variations in cell number and cell subpopulation distribution due to individual experimental variation. Therefore, it is important to recognize that global changes in transcript expression in response to mechanical stretch and/or BIRB796 treatment represent changes in the transcriptional profiles of both CM and non-CM. While some of the important adaptive responses are likely due to CM-autonomous regulatory cascades, the role of CM–non-CM coupling and the adaptive responses of myofibroblasts and other cell types within cardiac tissues is likely to have important biologic effects (Tulloch et al. 2001; Kobayashi et al. 2008; Thompson et al. 2011; Ongstad and Gourdie 2012; Zhang et al. 2012). Additional experiments are required to identify the cell lineage autonomous and nonautonomous effects of mechanical conditioning with ECT.

Our use of ECTs as the source for cardiac tissue transcript data is certainly a more simplified model than generating transcriptional data from intact ventricular myocardium (Gaborit et al. 2007; O'Brien et al. 2012; Gennebäck et al. 2013). For this study, we used a single vendor's gene transcript profiling chip and then selected a subset of altered transcripts for validation by q-PCR. After we discovered the large number of distinct transcripts altered in response to mechanical stretch and to BIRB796 treatment we selected a small subset of transcript for validation of changes in expression by q-PCR. We used this approach simply to validate the general accuracy of changes in transcript expression from the microarray data. However, substantial additional work is required to validate the role of individual molecular pathways in the transduction of mechanical stretch or BIRB796 treament into the biologic responses.

Perhaps most importantly, the analysis of single time-point transcript expression levels does not reflect the dynamic interactions between multiple regulatory mechanisms including miRNA and TF regulation of expression, variable rates of translation, and protein complex stabilization, activation, and degradation. Thus, this study provides an initial insight into the changes in gene expression noted in ECTs that may reflect multiple regulatory and adaptive cascades.

### Future directions

Our current study confirms the ability to generate genome-wide transcript expression data in ECTs during in vitro maturation and in response to increased mechanical loading and/or p38MAPK inhibition with BIRB796. Future experiments in ECTs are required which focus on selected regulatory networks that transduce the response to mechanical and/or metabolic load from stimulus to altered gene expression for defined regulatory molecules, such as Akt1 indicated by the IPA analysis and as has been completed for GATA-4 (Tenhunen et al. 2004) or FoxO (Sengupta et al. 2011).
